# Darbepoetin alfa is more potent *in vivo* and can be administered less frequently than rHuEPO

**DOI:** 10.1038/sj.bjc.6600506

**Published:** 2002-08-12

**Authors:** J Egrie, J Browne

**Affiliations:** Amgen Inc., Amgen Center Drive, MS 27-4-A, Thousand Oaks, California, CA 91320, USA

## Abstract

doi:10.1038/sj.bjc.6600506 www.bjcancer.com

© (2002) Cancer Research UK

## Sir

The development of darbepoetin alfa (NESP, ARANESP™) was an outgrowth of basic research directed towards elucidating those structural features that control the *in vivo* biological activity of erythropoietin (EPO). As described in our study, this research demonstrated that serum clearance is the primary determinant of EPO *in vivo* biological activity, and that serum clearance can be manipulated by changing the proportion of sialic acid-containing carbohydrate. Molecules having a higher proportion of sialic acid-containing carbohydrate have a longer serum half-life and thereby a higher *in vivo* biological activity. These principles, gleaned from natural sequence EPO, were applied and extended to specifically engineer a new molecule, NESP. Our review article (Egrie and Browne, 2001) summarized the basic research leading to the design of NESP and summarized the results of comparative pharmacodynamic studies of NESP and rHuEPO.

As we stated in our article, the relative potency of NESP and rHuEPO were determined in a normal mouse animal model in which both molecules produced dose-dependent increases in hematocrit. These studies were performed using three different routes of administration and three different dosing frequencies. For each route, frequency, and test article, multiple doses were tested which covered the entire dose response range. The results of these experiments were used to construct relative potency plots (log dose response curves). The relative potency was then determined as the ratio of equieffective doses of each from their respective graded dose–response relations (Tallarida and Murray, 1987). When data from all studies were combined (nine experiments using 1185 animals), NESP was determined to be 3.6-fold more potent than rHuEPO for each route of administration when given three times per week. That is, it takes 3.6-fold more rHuEPO to obtain the same biological response as NESP. As a corollary, when equimolar doses of NESP and rHuEPO are compared, rHuEPO produces a lower biological response, as is illustrated in our Figure 5 and noted in paragraph 3 of Dr Malonne's letter. Similarly, when NESP and rHuEPO were each administered once weekly, NESP was determined to be 13-fold more potent than rHuEPO. Relative potency plots were also used to compare once weekly with thrice weekly administration for each molecule. When rHuEPO was administered once weekly, an ∼15-fold higher total weekly dose was required to achieve the same biological response as when rHuEPO was administered thrice weekly. In contrast, for NESP only a 4-fold dose increase was required for once versus thrice weekly administration. As we stated in our article, due to the pharmacokinetic differences between NESP and rHuEPO (NESP has a 3-fold longer serum half-life than rHuEPO in animals and man) no one number can be used to express the relative potency difference between the two molecules. The relative potency of NESP and rHuEPO will necessarily change as a function of the dosing interval. Longer dosing intervals will lead to greater observed potency advantages for NESP.

As we cautioned in our review, one should be careful not to extrapolate the findings from an animal model to a human clinical setting. We cited the findings (Macdougall, 1998) from the first study of NESP in naïve dialysis patients, which did not show a difference in the NESP dose required once versus thrice weekly, and we suggested that the differences in erythrokinetics and red cell lifespan between the two species could account for this difference. Results from phase III clinical studies in chronic renal failure patients, however, confirm that the significantly longer serum half-life of NESP confers the clinical advantage of less frequent dosing compared with rHuEPO. In a crossover clinical study in dialysis patients, 97% of patients whose baseline rHuEPO dose frequency was two or three times per week, were successfully maintained on the same total weekly dose of NESP given once weekly or less frequently. In addition, in this study 95% of patients whose baseline rHuEPO therapy was once weekly were successfully maintained on NESP given once every other week (Vanrenterghem *et al*, 1999). Maintenance of Hgb level, weekly dose requirement and the frequency of dose changes in the NESP and rHuEPO groups were similar regardless of route of administration, even among NESP patients dosed once every other week. In the oncology setting, rHuEPO can be administered as infrequently as once a week (Gabrilove *et al*, 2001). In contrast, for NESP in the oncology setting, studies have clearly demonstrated that Hgb levels could be maintained when NESP was administered as infrequently as once every three or four weeks (Kotasek *et al*, 2000, 2002; Smith *et al*, 2002). In total, these studies indicate that NESP provides the clinical benefit of less frequent dosing.

As Dr Malonne noted, the five amino acid changes made to design NESP could have consequences on the structure of the molecule. Although biophysical characterisation of NESP was not the subject of our review, we report here that the tertiary structures of the NESP and rHuEPO polypeptides are virtually indistinguishable by a variety of different tests. However, as stated in our article, since any change in the primary, secondary or tertiary structure of a protein may be recognised by the immune system of treated patients, all patients in clinical studies have been carefully monitored for the development of an immune response. Significantly, in all clinical trials, involving over 10 000 patients, there has been no evidence of antibody formation.
